# Magnitude and associated factors of Intestinal Parasitosis and Tuberculosis among Tuberculosis suspected patients attending Kuyu General Hospital, North Shewa, Oromia, Ethiopia

**DOI:** 10.1371/journal.pntd.0010120

**Published:** 2022-01-10

**Authors:** Sahilu Tesfaye, Biruk zerfu, Kassu Desta

**Affiliations:** Department of Medical Laboratory Sciences, College of Health Sciences, Addis Ababa University, Addis Ababa, Ethiopia; NIH-National Institute for Research in Tuberculosis-ICER, INDIA

## Abstract

**Background:**

Intestinal parasites and Tuberculosis (TB) co-infection is a major public health problem. The parasitic infection suppresses the cell mediated immunity that protects tuberculosis. Helminthes-induced immune modulation promotes progression to active tuberculosis. However, there is paucity of evidences on the intestinal parasites-tuberculosis co-infection in Ethiopia. This study explores the magnitude and associated factors of intestinal parasitic infection and TB among suspected pulmonary Tuberculosis (PTB) patients.

**Methodology:**

A cross-sectional study design was conducted in Kuyu General Hospital from December 2019—March 2020. The socio-demographic data and associated factors were collected by structured questionnaire and then spot-spot sputum and fresh stool samples were collected following standard guidelines and were processed. Descriptive analysis was conducted and reported in frequency and percentage. Bivariate analysis was computed and a multivariable analysis was conducted to provide an adjusted odds ratio (AOR). P-value <0.05 at 95% confidence interval was considered as statistically significant.

**Results:**

The burden of intestinal parasites was 20.2% (49/ 242) and 6.1% (20/ 242) of them were helminths infections and 14.1% (29/ 242) were protozoa infections. Of 242 patients, 14.9% (36/242) were sputum smear-positive for acid fast-bacilli. Of 36 smear positive patients, 9(25%) had TB–intestinal parasites co-infection. Dwelling in rural areas and having untrimmed fingernails were statistically significantly associated with intestinal parasites. Having a contact history of Tb patients was significantly associated with pulmonary tuberculosis.

**Conclusions:**

The magnitude of intestinal parasites and TB among PTB suspected patients were high. Hookworm infection was the predominant helmenthic infection. It is important to consider screening TB patients for intestinal parasites and treat co-infection properly.

## Author summary

TB and helminthic infections co-occur exists, particularly in areas where poverty persists. Both diseases can affect the same individual and exacerbate the course of diseases. There are a few reports about prevalence of intestinal parasites, TB and TB co-infection, which varies from place to place due to geographical distribution and life style. Hence, we tried to reveal intestinal parasitosis-tuberculosis co-infection among PTB suspected patients. We also tried to address associated factors for intestinal parasitosis and tuberculosis. Since the burden of co-infection causes a major public health threat, health care providers should screen and request all necessary tests used to diagnose in order to identify the co-infection, treat TB patients for co-infection.

## Introduction

Tuberculosis (TB) is a major public health threat [1,2]. It is one of the top 10 causes of mortality with an estimated 10 million new cases each year worldwide [3].The TB disease has been challenging in sub-Saharan African countries due to inadequate health infrastructures and scarce resources [4]. Intestinal Parasitic Infections (IPIs) have been impacting the world public health, especially among socio-economically disadvantaged populations. Globally nearly 3.5 billion people have been infected and caused morbidity in an estimated 450 million people by these intestinal parasites [5].

The case burden of TB is higher in Ethiopia (177/100,000 population) than that of the average case burden of TB worldwide (133/100,000) [3,6]. Social and behavioral factors including smoking, drinking alcohol, drinking raw milk, having a family history of tuberculosis, living in poor ventilated and inadequate size room, and rural residence are among risk factors hastening the disease progress [7–9]. On the other hand, an estimated 81 million Ethiopian populations are dwelling in endemic areas of the IPIs [10]. The TB and IPIs are found co-infecting the community to exacerbate the course of diseases [11]. This is supported by the findings of 32% of patients admitted due to TB intestinal parasitosis and 29% of individuals diagnosed positive for TB from the community developed intestinal parasitic infections[12]. Therefore, the co-existence of these two infectious agents is a public health issue [2].

The immune response of patients with Tb and intestinal helminths co-infection was decreased against *Mycobacterium tuberculosis* (MTB) [5]. The helminth infection induces strong Th2 anti-inflammatory responses. Due to the fact that, helminths are able to induce strong CD4+ T-helper 2 (Th2) anti-inflammatory responses through induction of variety of cytokines such as: IL-4, IL-5, IL-9 and IL-13 and increased levels of eosinophils and circulating IgE antibodies, which may suppress Th1 immune response [2,13]. However, switching from Th_1_ toward Th_2_ during parasitic infection causes the down-regulation of T-helper 1 (Th1) cells which assist in protection against TB [14]. The control of *M*. *tuberculosis* infection requires pro-inflammatory Th_1_ (IL-12, IFN-γ and TNF-α) [11,15].

Since chronic helminths infection can affect the ability of the host to protect against mycobacterial infections [10], the current vaccine against TB, Bacillus Calmette Guerin (BCG), has poor efficacy against adult PTB that might be due to helminth infection modulates the immune response by increasing regulatory T cells activity [1,15].

The immune-modulations by both infections enhance survival, multiplication and dissemination of *M*. *tuberculosis* to develop active Tuberculosis. Such independent immune-modulation determines the consequence of both infections [16,17].

Several studies have reported an increased magnitude of intestinal parasites among TB patients [13,18]. The study conducted in India showed that the co-infection of TB with intestinal parasites was 27.11% [18]. In Ethiopia, a study conducted in Addis Ababa and Arbaminch estimated the infection rate of intestinal parasites among PTB patients to 22% and 26.3% respectively [16,19].

Even though the co-infection of TB and intestinal parasites hastens progression of the disease, increases morbidity in PTB patients, increases the complexity of control and prevention on Tb and parasitic diseases [1,2], In Ethiopia there are a few reports about magnitude of intestinal parasitosis, TB and TB co-infection. As a result, adequate information on intestinal parasites with Tuberculosis in such cases is scarce.

However, adequate studies enable us to create strong surveillance system, policy development or updating which makes us alert in order to prevent and control risk of mortality and morbidity, reduce medical care cost and loss of productivity caused by this complex co-infection.

Therefore, this study aimed to assess the magnitude and associated factors of intestinal parasitic infection and TB among suspected TB patients to fulfill the observed Discrepancy.

## Materials and methods

### Ethics statement

The study was obtained ethical approval from Research and Ethical Review Committee of the Department of Medical Laboratory Sciences, College of Health Science, Addis Ababa University. A written informed consent was obtained from each participant prior to enroll to the study and from parents or guardians for children. Children from 12–17 years also gave assent. The information obtained from participants was kept confidential and used only for the purpose of the achieving the objectives. Data recording and storing was done by principal investigator and carefully stored under key and lock. The participants were clearly explained to provide information that would improve the care for TB suspected patients.

### Study design and study area

A cross-sectional study design was conducted to determine the magnitude and associated factors of intestinal parasitosis and tuberculosis among PTB suspected patients attending Kuyu General Hospital, Ethiopia from December 2019 to March 2020. It provides service for about 245,000 catchment area populations living in Kuyu Woreda and four neighboring woredas (Wore jarso, Degem, Hidabu Abote and Gundo meskel). Kuyu district is found in North Shewa zone of Oromia Region and distant 150 Km to the North from the capital Addis Ababa. The administrative center of Kuyu is Gerbe Guracha town that has latitude and longitude 9^0^48’N, 38^0^24’E and Elevation between 2515 and 2547 meters above sea level. Gerba Guracha town is located on the road from Addis Ababa to Grand Renaissance Dam (GRD) that has high population transit.

### Eligibility criteria and sampling

**Fig 1:**
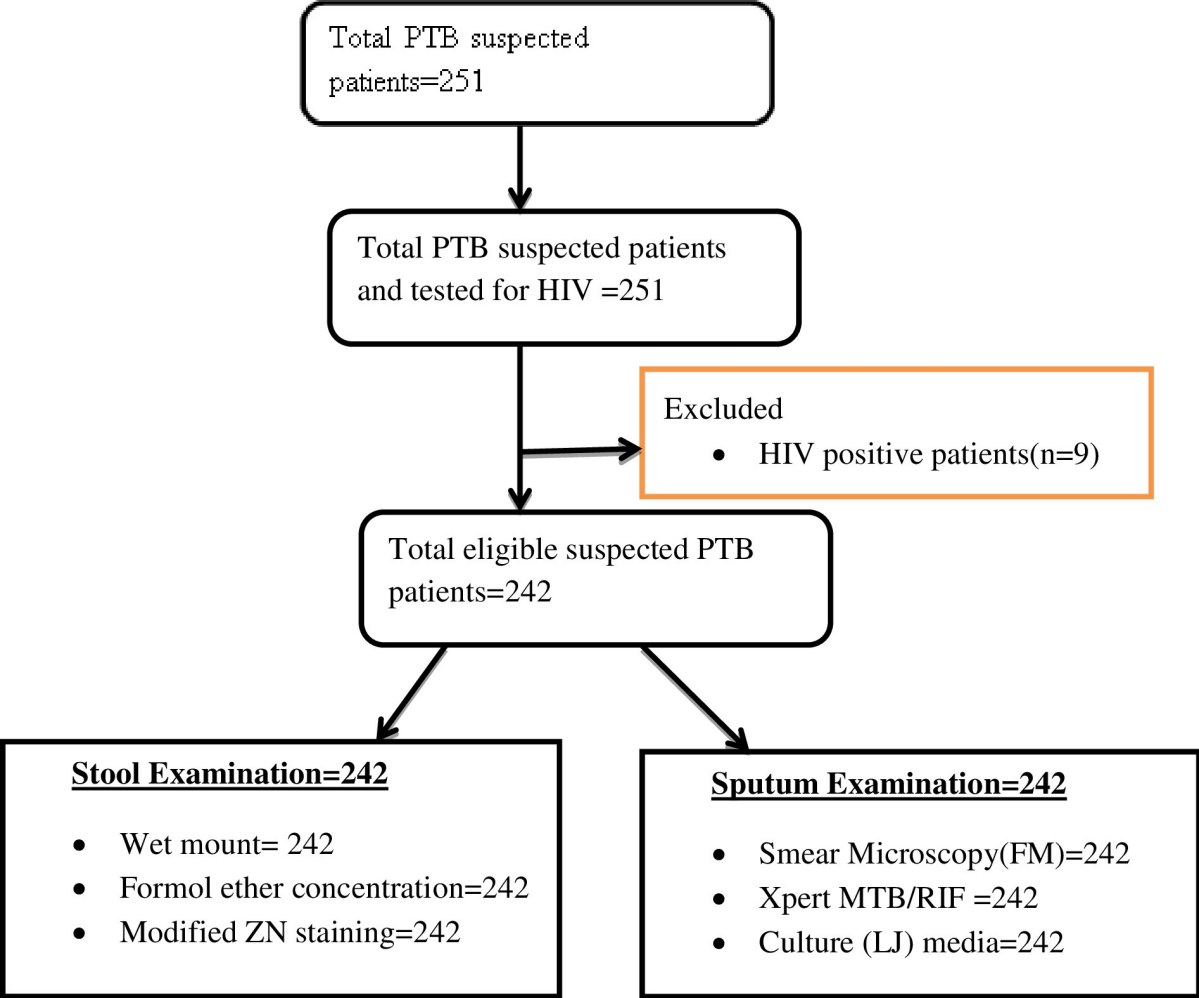
Flow chart for enrollment and data collection procedure for PTB suspected patients in Kuyu General Hospital, Ethiopia, 2020.

All Patients suspected to PTB or having cough of two weeks or more who accepted the informed consent were included in study population. A patient was considered as Suspected to PTB when that individual had cough of two weeks or more and Signs and symptoms of active TB disease, but his/her medical evaluation was not completed. However, HIV positive individuals, clients who had already started anti-TB treatment and participants who took anti helmenthic drugs during the two weeks before specimen collection were excluded (Fig 1). The sample size was calculated as per formula for calculating sample size using epi Info statistical and the precision around a proportion. To calculate sample size, we employed 95% confidence interval, 5% degree of precision around the mean and prevalence from similar previously conducted study, p = 19.6% [20].

Therefore, an estimated 242 individuals were enrolled in the study. Convenient sampling method was used to recruit the participants. The principal investigator visited all the outpatient and inpatient departments and recruited the data enumerators.

### Questionnaire

Using pre-tested questionnaire, the socio-demographic information including gender, age, marital status, occupation, educational status, residence, Anthropometric parameter (BMI), behavioral characteristics, history of previous hospitalizations and medical treatments and environmental conditions (Latrine usage, Hand washing after defecation, Water source, Raising livestock, Eating raw meat and Washing Cloth habit) were collected from the study participants by trained and fluently speakers of local language data collectors. The questionnaire was standardized by 5% pre-testing conducted in Tulu bolo General Hospital, Southwest Shewa, Ethiopia two weeks prior the actual data collection. Based on pre-test, the questionnaire was reviewed and formatted. The supervision was also conducted during data collection. The collected data was checked for completeness.

### Collection of samples

Clinical data were obtained from patients’ folder and from results of laboratory tests. Smear microscopy, Xpert MTB/RIF assay, culture and stool examination were conducted by experienced, trained and senior laboratory personnel.

BMI was measured as weight in kilograms divided by the square of height in meters (kg/m2) and was interpreted using standard categories as Underweight, Normal, Overweight and Obese when Below 18.5, 18.5–24.9, 25.0–29.9, 30.0 and above respectively[21].

#### Sputum sample collection

Sputum was collected using appropriately labeled clean, sterile, disposable containers. Screw caps must fit tightly to avoid unnecessary leakage of sample. We were use falcon tube for Genexpert sample collection. Gastric aspiration or sputum induction procedure was used to obtain a specimen when a patient cannot cough up adequate sputum by consulting the physicians. Two sputum samples, spot- spot sputum samples for Fluorescent microscope (FM) with in half to an hour after collection and a morning sample for *Xpert MTB/RIF assay* and culture were collected.

#### Stool sample collection

Uncontaminated a single fresh sample per every patient was collected, transported and examined within 30 minutes of passage.

### Laboratory analysis

#### AFB sample processing

For smear microscopy, a smear prepared from purulent sample was flooded with auramine O, and then it was flooded with 0.5% acid alcohol. Finally the slides were flooded with 0.5% potassium permanganate and examined by FM [22].

Acid fast bacilli (AFB) was examined by total 400X magnification and described using the World Health Organization grading scale as: No AFB observed (No AFB in one length), scanty (3–24 AFB in one length), +(1–6 AFB in one field),++ (7–60 AFB in one field) and+++ (> 60 AFB in one field)[22].

For *Xpert MTB/RIF assay*, decontaminated sputum samples containing sample reagent that recommended at a 3 to1 ratio for sputum pellets were incubated at room temperature for 15 minutes to reduce the viability of *M*. *tuberculosis* then manually transferred to the cartridge that was loaded into the *GeneXpert* instrument. Then test started on to process sample and finally result was read and interpreted [17,23].

For culture, samples were processed using a mucolytic agent, N-acetyl-L-cysteine (NALC) and NaOH, decontaminating agent. Sodium citrate was added on NALC to stabilize and the phosphate buffer (PH 6.8) neutralized the NaOH. The sample centrifuged at 3000×g for 15 minutes to sediment mycobacteria. The suspended sediment was inoculated in Lowenstein Jensen (LJ) Media. The result was observed weekly and reported negative when no growth for eight weeks [22].

### Stool sample processing

Fresh fecal specimens with appropriate volume (2–3 gm) were properly collected in labeled clean, wide-mouth containers with tight fitting lids before taking anti-helmenthic drugs and delivered to parasitology section of Kuyu Hospital for processing [24].

Direct wet smear was prepared by mixing a small amount (50gm) of fresh, unpreserved stool with a drop of 0.85% saline and then was examined under a 22- by 22-mm cover slip. The entire coverslip was examined with the low-power (10x) objective and suspicious objects examined at 40x objective [24].

Moreover, formol-ether concentration was done using, 1 gram of stool sample that was mixed in 7ml of 10% formol saline and it was kept for 10 minutes to fix it. It was then filtered through wire gauze. The filtrate was added to 3 ml of ether and it was centrifuged at 2000 rpm for 2 minutes. It was allowed to settle. The supernatant was discarded and a wet mount was made of the deposit to examine using 10 and 40 times objectives for the presence or absence of parasites [25]. In addition, the second smear was prepared for modified zeehel Nelson stain (ZN).

In modified zeehel Nelson stain, a smear prepared from formol-ether concentration was flooded with strong carbol fuchsin, decolorized using 5% sulfuric acid in alcohol, and counter stained with methylene blue, to recognize the lipids in the wall of Cryptosporidium oocysts. The stained specimen was examined using oil immersion objectives (100) [24].

### Data quality

Structured questionnaire prepared for face-to-face interview was translated to local language and re-translated back to keep consistency and was pre-tested on patients who did not enrolled into the study. Specimens were handled with suitable collection containers and with complete patient information. All fresh stool specimens were examined within 30 minutes of collection or in the case of delay we used appropriate preservatives to preserve protozoan morphology and to prevent continued development of various helmenth eggs and larvae [24]. Preventive maintenance for instruments was performed and quality control materials were processed properly prior patient samples processing. Principles and procedures of the tests were strictly followed. Data collectors and a supervisor were trained for three days concerning objective of the study, data quality and overall process of data collection before deployment to data collection. All slides examined by the first microscopist were verified by others two microscopist for quality control. The examinations were performed by four trained experienced laboratory technologists. Attention was paid to prevent cross contamination from a heavily acid-fast positive specimen to other negative samples. Finally, the result was reported after checked and verified.

### Data analysis

Data was entered into EPI info version 7 and was exported to SPSS version 23. Descriptive analysis like socio-demographic characteristics was conducted by SPSS and finally presented accordingly by text, tables and figures.

Bivariate analysis was computed for categorical parameters. A multivariate analysis was conducted by fitting a multiple logistic regression model on the independent variables that were significant at, P <0.25 which provide adjusted parameter estimates (AOR). A two sided *P*-value < 0.05 at 95% confidence was considered as statistically significant.

## Results

### Socio-demographic characteristics of study participants

A total of 242 (122 male, 120 female) of PTB suspected patients were included in this study. The age of the participants was ranged from 1 to 90 years with the mean age of 38.9(SD = ±19.8) years and majority 149(61.6%) of the PTB suspected patients were between 20–59 years. Among all study participants, 172(71.1%) were rural dwellers and 128(52.9%) were not able to read and write. Out of 242 study participants, 159(65.7%) were employed to different occupation whereas the rest 83(34.3%) were non-employed.

For the case of marital status, 173(71.5%) were married and 69(28.5%) were single. On the other hand, the behavioral characteristics of the habit of latrine usage, shoe wearing, swimming, and hand washing before and after meals were accounted 177(73.1%), 232(95.9), 6(2.5%) and 240(99.2%) of these TB suspected patients respectively.

Majority of the participants, 157(64.9%), were reported as they wash their hands after defecation; of them 153(63.2%) individuals were used to wash only with water whereas 5(2.1%) were with both water and soap. For the response of water source, 140(57.9%) participants were used water from river and 15(6.2%) were washed their cloths using river water (Table 1).

**Table 1:** Socio-demographic and behavioral characteristics of the PTB suspected patients attending Kuyu General Hospital, North Shewa, Oromia, Ethiopia, 2020(n = 242).

Variables		Frequency	Percent (%)
Age group			
	<10	15	6.2
	10–19	33	13.6
	20–59	149	61.6
	≥60	45	18.6
Sex			
	Male	122	50.4
	Female	120	49.6
Residency			
	Urban	70	28.9
	Rural	172	71.1
Education status			
	No education	128	52.9
	Primary	55	22.7
	Secondary	42	17.4
	Diploma and above	17	7
Occupation			
	Unemployed	83	34.3
	Employed	159	65.7
Marital status			
	Single	69	28.5
	Married	173	71.5
BMI	<18.5	79	32.6
	18.5–24.9	155	64
	≥25	8	3.3
Latrine usage			
	Yes	177	73.1
	No	65	26.9
Swimming habit			
	Yes	6	2.5
	No	236	97.5
Shoe wearing habit			
	Yes	232	95.9
	No	10	4.1
Bathing habit			
	River	14	5.8
	Home	129	53.3
	Home and River	99	40.9
Hand washing before meal			
	Yes	240	99.2
	No	2	0.8
Hand washing after defecation			
	Yes	157	64.9
	No	85	35.1
Hand washing after defecation			
	No hand washing	84	34.7
	Water	153	63.2
	Water and soap	5	2.1
Water source			
	River	140	57.9
	Pipe	102	42.1
Washing Cloth habit			
	Home and River	91	37.6
	River	15	6.2
	Home	136	56.2
Fingernail status			
	Trimmed	158	65.3
	Untrimmed	84	34.7
Un washable vegetable			
	Yes	85	35.1
	No	157	64.9
Eating raw meat			
	Yes	143	59.1
	No	99	40.9
Raising livestock			
	Yes	167	69
	No	75	31

On the other hand, from the analysis of behavioral characteristics, the BCG vaccination, having contact history of TB patients and smoking cigarette in the past one year were 19(7.9%), 92(38%) and 14 (5.8%) respectively (Table 2).

**Table 2:** Behavioral characteristics of the PTB suspected patients attending Kuyu General Hospital, North Shewa, Oromia, Ethiopia, 2020(n = 242).

Variables		Number of respondents	Percent (%)
BCG vaccination			
	Yes	19	7.9
	No	223	92.1
Having contact history of TB patient			
	Yes	92	38
	No	150	62
Having a past history of PTB			
	Yes	23	9.5
	No	219	90.5
Smoking cigarette in the past one year			
	Yes	14	5.8
	No	228	94.2
Drinking alcoholic beverage			
	Yes	96	39.7
	No	146	60.3
Imprisoned in the past one year			
	Yes	7	2.9
	No	235	97.1
Drinking Raw Milk			
	Yes	143	59.1
	No	99	40.9

### Magnitude of intestinal parasites and tb among ptb suspected patients

**Fig 2:**
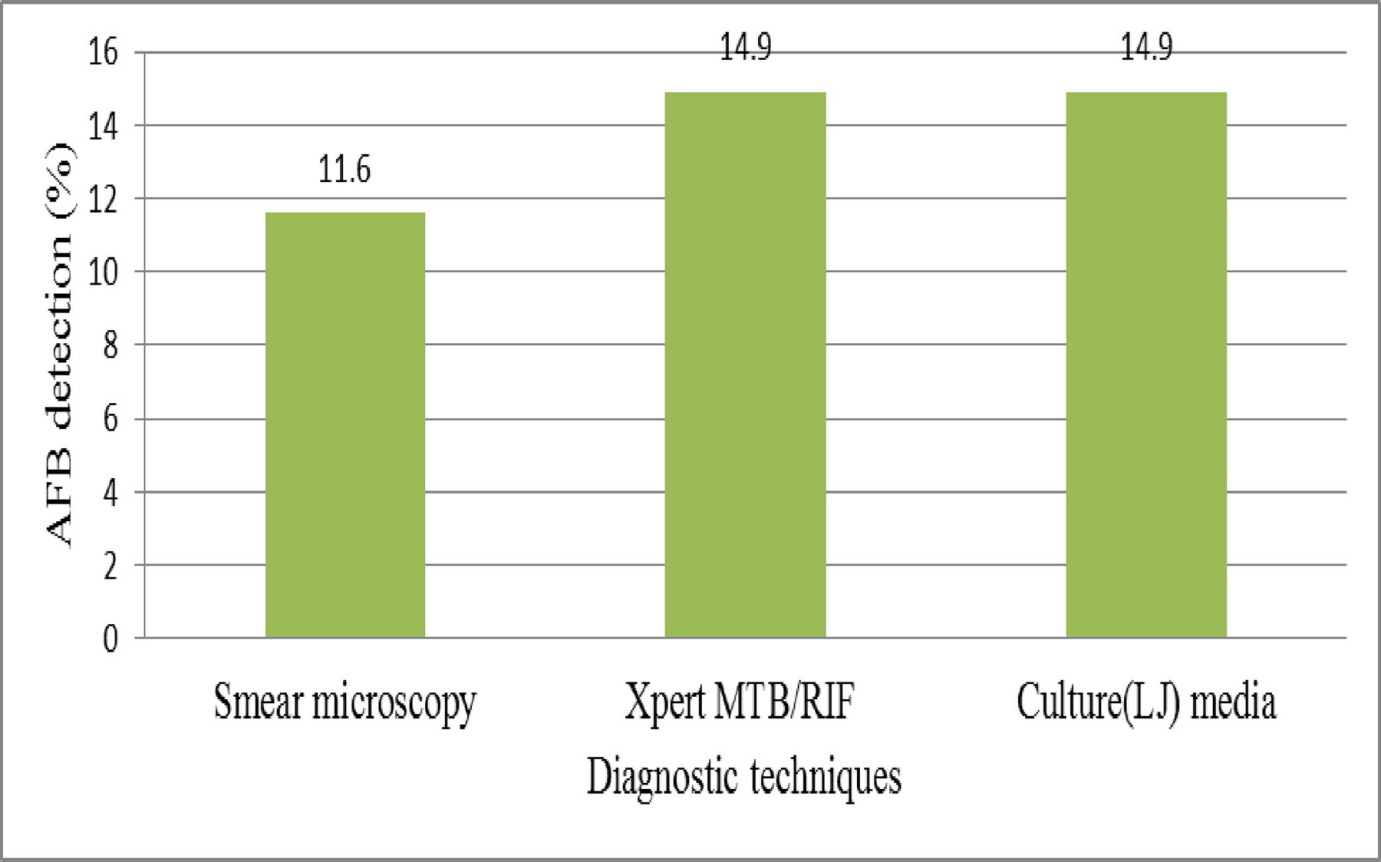
Magnitude of tuberculosis among PTB suspected patients in Kuyu General Hospital, 2020 (n = 242).

Among 242 participants PTB was detected in 36(14.9% %; 95% CI: 10.7–19.4) patients (Fig 2). However, no Rifampicin resistance TB (RR-TB) was detected.

On the other hand, among 242 PTB suspected patients, intestinal parasites were detected in 49 (20.2%; 95% CI: 15.3–25.6) individuals. The infection intestinal parasites among females and males were 20.8% (25/120) and 19.7% (24/122) respectively. Maximum (32/149, 21.5%) of those having IPIs were in the age group of 20–59 years followed by age group greater than 60 years (9/45, 20%)(Table 3).

**Table 3:** Associated factors for intestinal parasitosis among PTB suspected patients attending Kuyu General Hospital, North Shewa, Oromia, Ethiopia, 2020(n = 242).

Variables		Intestinal parasite infection (%)	COR (95% CI)	P-value	AOR(95% CI)	P-value
Age group					-	
	<10	3/15(20)	1.00			
	10–19	5/33(15.1)	0.74(0.15–3.4)	0.67		
	20–59	32/149(21.5)	1.1(0.29–4.1)	0.89		
	≥60	9/45(20)	1(0.23–4.3)	0.99		
Sex					-	
	Male	24/122(19.7)	0.93(0.50–1.74)	0.82		
	Female	25/120(20.8)	1.00			
Residency						
	Urban	6/70(8.6)	1.00			
	Rural	43/172(25)	3.56(1.44–8.79)	0.006	**3.19(1.27–8.0)**	**0.013** *
Education status					-	
	No education	28/128(21.9)	1.24(0.66–2.33)	0.505		
	Literate	21/114(18.4)	1.00			
Occupation						
	Unemployed	12/83(14.4)	0.55(0.27–1.14)	0.108	0.61(0.29–1.27)	0.19
	Employed	37/159(23.3)	1.00			
Marital status					-	
	Single	12/69(17.4)	1.00			
	Married	37/173(21.4)	1.29(0.63–2.66)	0.486		
BMI	<18.5	18/79(22.8)	1.26(0.65–2.4)	0.5		
	18.5–24.9	31/163(19)	1.00			

Note

*P<0.05, statistically significant association; Confidence intervals that do not overlap 1 are shown in bold.

**Abbreviations:** AOR = adjusted odds ratio; CI = Confidence interval; COR = crude odds ratio

**Fig 3:**
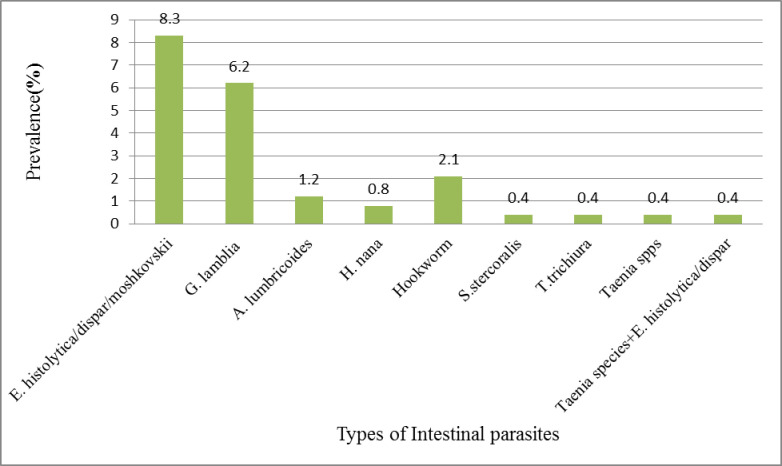
Parasitic infection of participants in a study of intestinal parasite among PTB patients in Kuyu General Hospital, 2020 (n = 242).

While analyzing the different type of IPIs among PTB suspected patients, Out of a total 242 patients, 14(5.8%) had the helmenths infection and 35(14.5%) had protozoal infection. *Entamoeba histolytica/dispar/moshkovskii* was the most common protozoa infection accounted to 20(8.3%) followed by Giardia lamblia with infection rate of 15(6.2%). Mixed infection (Taenia species and *E*. *histolytica/dispar/moshkovskii)* was found in one patient (0.4%). (Fig 3)

### Co-infection of intestinal parasites and PTB among PTB Suspected patients

**Fig 4:**
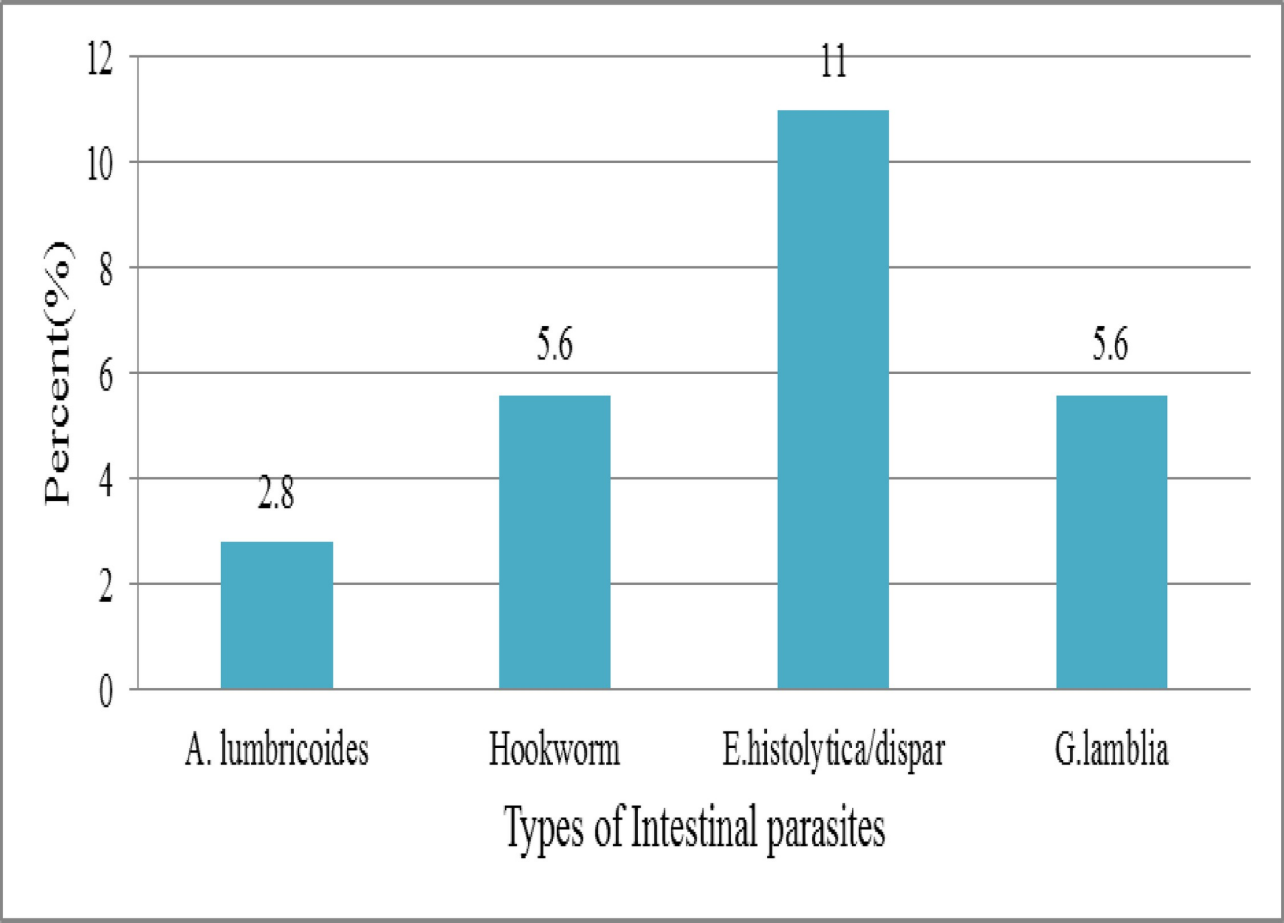
Intestinal Parasite-Tuberculosis co-infection among AFB positive, Kuyu General Hospital, North Shewa, Oromia, Ethiopia, 2020(n = 36).

Out of 36 AFB positive participants 25% (9/36) patients had IPI and TB co-infection. On the analysis of co-infection, two helminths and two protozoa species were detected. The helminths were estimated to 8.3% (3/36). Hookworm (2/36, 5.6%) was the most predominant helminthic infection among smear positive patients followed by *Ascaris lumbricoides* (1/36, 2.8%) (Fig 4).

**Fig 5:**
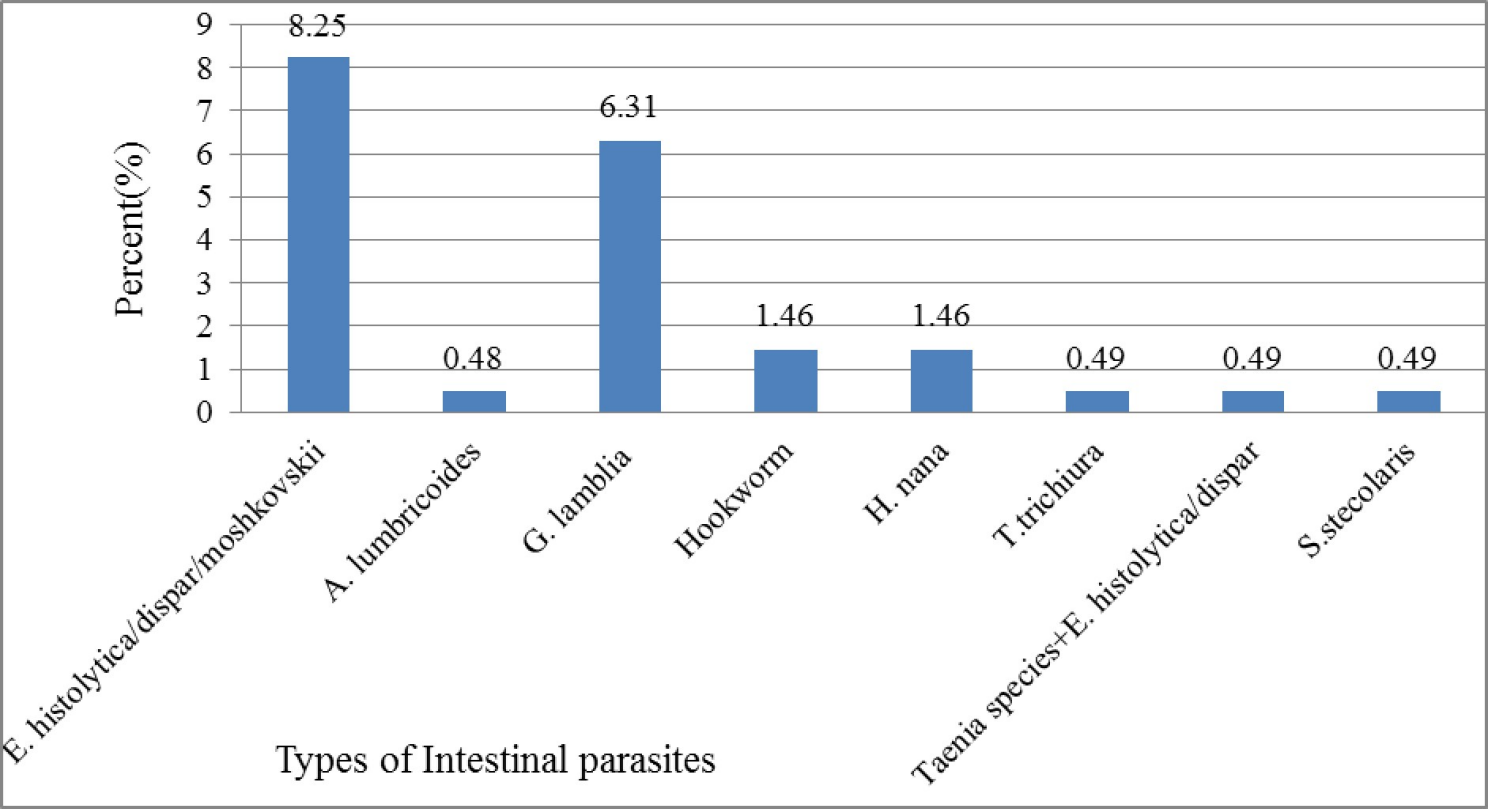
Intestinal parasitic infections among smear negative individuals in Kuyu General Hospital, 2020 (n = 206).

However, out of a total 206 smear negative individuals, intestinal parasites were detected among 19.4% (40/206) individuals including 14.6% (30/206) protozoa infections and 4.9% (10/206) helminths infections. Hookworm was accounted to 3/206(1.46%) and *A*. *lumbricoides* was accounted to 1/206(0.48%) (Fig 5).

### Factors associated with intestinal parasites among PTB Suspected patients

The socio-demographic characteristics and behavioral characteristics were analyzed by bi-variable logistic regression and obtained four factors associated with IPIs at p < 0.25 including the residency area, occupation of the respondents, eating raw meat and fingernails status. These variables (p < 0.25) were inserted into multivariable logistic regression analysis (Tables 3 and 4).

**Table 4:** Associated factors for intestinal parasitosis among PTB suspected patients attending Kuyu General Hospital, North Shewa, Oromia, Ethiopia, 2020(n = 242).

Variables		Intestinal parasite infection (%)	COR (95% CI)	P-value	AOR(95% CI)	P-value
Latrine usage					-	
	Yes	36/177(20.3)	1.00			
	No	13/65(20)	0.98(0.48–1.99)	0.95		
Swimming habit					-	
	Yes	1/6(16.7)	0.78(0.09–6.86)	0.82		
	No	48/236(20.3)	1.00			
Shoe wearing habit						
	Yes	46/232(19.8)	1.00		-	
	No	3/10(30)	1.73(0.43–6.96)	0.44		
Bath habit					-	
	Home	20/99(20.2)	1.00	0.99		
	Home and River	26/129(20.2)	1.0(0.52–1.92)			
	River	3/14(21.4)	1.08(0.27–4.23)			
Hand washing after defecation					-	
	Yes	30/157(19.1)	1.00			
	No	19/85(22.3)	1.22(0.64–2.33)	0.55		
Hand washing after defecation					-	
	No hand washing	19/84(22.6)	1.15(0.12–10.9)	0.90		
	Water	29/153(19)	0.94(0.10–8.76)	0.96		
	Water and soap	1/5(20)	1.00	0.84		
Water source					-	
	River	21/102(20.6)	1.04(0.55–1.96)	0.91		
	Pipe	28/140(20)	1.00			
Washing Cloth habit					-	
	Home	30/136(22)	1.00	0.41		
	Home and River	18/91(19.8)	0.87(0.45–1.68)			
	River	1/15(6.6)	0.25(0.03–1.99)			
Fingernail status					-	
	Trimmed	24/158(15.2)	1.00			
	Untrimmed	25/84(29.8)	2.37(1.25–4.48)	0.008	**2.31(1.12–4.47)**	**0.012** *
Un washable vegetable					-	
	Yes	16/85(18.8)	0.87(0.45–1.7)	0.69		
	No	33/157(21)	1.00			
Eating raw meat						
	Yes	25/143(17.5)	0.66(0.35–1.24)	0.20	0.65(0.34–1.25)	0.20
	No	24/99(24)	1.00			
Raising livestock					-	
	Yes	35/167(21)	1.16(0.58–2.30)	0.682		
	No	14/75(18.7)	1.00			
Sharing the same house					-	
	Not raising the livestock	14/75(18.7)	1.00			
	Yes	9/46(19.5)	1.06(0.42–2.69)	0.90		
	No	26/121(21.5)	1.19(0.58–2.46)	0.63		

Note

*P<0.05, statistically significant association; Confidence intervals that do not overlap 1 are shown in bold.

**Abbreviations:** AOR = adjusted odds ratio; CI = Confidence interval; COR = crude odds ratio;—not done

Finally, the overall factors associated with intestinal parasites were identified after adjusting for possible confounders by multivariate analysis. Dwelling in rural area (AOR = 3.19, 95% CI: (1.27–8.0) and untrimmed fingernails (AOR = 2.31, 95% CI: 1.12–4.47) were found statistically significantly associated with intestinal parasites (Tables 3 and 4). The odds of contracting intestinal parasitosis among people who were dwell in the rural area increases by a factor of 3.19 as compared to those who live in urban (AOR = 3.19, 95% CI: 1.27–8, p<0.013) (Table 3).

Similarly, compared to those who trimmed their fingernails, those who had untrimmed fingernail had higher odds of developing intestinal parasitosis (AOR = 2.31, 95% CI: (1.12–4.47, p < 0.012) (Table 4).

### Factors associated with tuberculosis among PTB Suspected patients

In analysis of bi-variable logistic regression for socio-demographic characteristics; Gender of the respondents and educational status were found to be associated with PTB with p-values less than 0.25 (p < 0.25).

While analyzing the logistic regression using behavioral factors, having contact history of TB patient and consuming alcoholic beverage were factors candidate to be analyzed by multivariable analysis (p < 0.25).

We adjusted for all factors including gender, educational status, consuming alcoholic beverage and having contact history of TB patient. We computed the multivariable logistic regression and obtained that having contact history of TB patient was statistically significantly associated with acquiring PTB. The odds of contracting PTB among participants who were having contact history of TB patients increases by a factor of 2.88 as compared to those who did not have TB contact (AOR = 2.88,95%CI:1.37–6, p<0.005)(Table 5)

**Table 5:** Multivariate analysis for PTB among PTB suspected patients attending Kuyu General Hospital, North Shewa, Oromia, Ethiopia, 2020(n = 242).

Variables		TB infection (%)	COR (95% CI)	P-value	AOR (95% CI)	P-value
Age group					-	
	<10	2/15(13.3)	1.00			
	10–19	6/33(18.2)	1.4(0.25–8.1)	0.67		
	20–59	25/149(16.8)	1.3(0.28–6.1)	0.73		
	≥60	3/45(6.7)	0.36(0.07–3.0)	0.43		
Sex						
	Male	22/122(17.8)	1.67(0.81–3.43)	0.17	1.6(0.74–3.3)	0.25
	Female	14/120(11.8)	1.00			
Residency					**-**	
	Urban	11/70(15.7)	1.00			
	Rural	25/172(14.5)	0.91(0.44–2)	0.81		
Education status						
	No education	14/128(10.9)	0.51(0.25–1.1)	0.07	0.61(0.29–1.23)	0.20
	Literate	22/114(19.3)	1.00			
Occupation					-	
	Unemployed	11/83(12.8)	0.82(0.38–1.76)	0.60		
	Employed	25/159(16)	1.00			
Marital status					-	
	Single	11/69(15.9)	1.00			
	Married	25/173(14.4)	0.89(0.41–1.93)	0.77		
BMI	<18.5	13/79(16.5)	1.2(0.57–2.5)	0.63		
	18.5–24.9	23/163(14.1)	1.00			
BCG vaccination					-	
	Yes	3/19(15.8)	1.00			
	No	33/223(14.8)	0.92(0.25–3.35)	0.90		
Having contact history of TB patient						
	Yes	22/92(23.9)	3.05(1.47–6.33)	0.003	**2.88(1.37–6.0)**	0.005*
	No	14/150(9.3)	1.00			
Having a past history of PTB					-	
	Yes	5/23(21.7)	1.68(0.58–4.8)	0.34		
	No	31/219(14.2)	1.00			
Smoking cigarette in the past one year					-	
	Yes	3/14(21.4)	1.61(0.42–6.08)	0.48		
	No	33/228(14.5)	1.00			
Drinking alcoholic beverage						
	Yes	18/96(18.8)	1.64(0.80–3.34)	0.17	1.49(0.72–3.11)	0.28
	No	18/146(12.3)	1.00			
Imprisoned in the past one year					-	
	Yes	2/7(28.6)	2.36(0.44–12.7)	0.31		
	No	34/235(14.5)	1.00			
Drinking Raw Milk					-	
	Yes	19/143(13.2)	0.74(0.36–1.5)	0.40		
	No	17/99(17.2)	1.00			

Note

*P<0.05, statistically significant association; Confidence intervals that do not overlap 1 are shown in bold.

**Abbreviations:** AOR = adjusted odds ratio; CI = Confidence interval; COR = crude odds ratio;—Not done

## Discussion

Intestinal parasites (IPs) and TB are major concerns in most developing countries to cause severe morbidity and affect most deprived communities. This study was intended to identify the magnitude and associated factors of intestinal parasitosis and TB among PTB suspected patients. The findings of the study revealed that the magnitude of intestinal parasites among tuberculosis suspected patients was 20.2%. The finding from this study was comparable with the study conducted in Ethiopia among active PTB patients in selected health centers in Addis Ababa (22%) [19].

Contrary, this finding is lower than study conducted in Arba minch (26.3%) [16], in Gonder, North-West Ethiopia (49%) [11], Pulmonary Tuberculosis Patients in India (27.11%)[18], systematic review and meta-analysis in Iran (26%)[26], Tanzania (29.5%)(12). This might be ascribed to many factors like de-worming or anti-helmenthic drugs administration specifically for young children at community level, Hygiene and environmental sanitation awareness provided by health extension workers and environmental health professionals to reduce contamination of soil, food and water by promoting the use of latrines and hygienic behavior. However, our finding is higher as compared to study conducted in rural China (14.9%) [6]. The existence of such variation may be explained by the difference in geographic location, socio-economic conditions, sample size, in the selection criteria of study participants; in our study the study population was PTB suspected patients, starting of anti-M*ycobacterium tuberculosis* treatment; difference in diagnostic methods should also be considered as a possible reason behind the disparity in the infection rates.

In this study, intestinal protozoa infections (14.5%) were the most frequently detected among TB suspected patients. This is consistent with study conducted in Addis Ababa, Ethiopia [19]. However, lower than study conducted in Arba minch, Ethiopia where *Ascaris lumbricoides* predominantly occur [16]. It is likely due to the fact that they used untreated water for drinking and for washing fruits and vegetables. In addition, handling of foods and drinking water with contaminated hands can cause protozoa infection.

The finding from our study revealed that the magnitude of intestinal helmenthiasis (5.8%) was seen among PTB infected patients. Similar result was reported from previous literatures [18,20,26]. This may be due to intestinal helminths infections tending to modulate the immune response cytokine profile to a T-helper type-2 response, which may cause T-helper type-1 imbalance. Chronic helminth infection may affect host immunity in TB and diminished cellular immune response against TB [15,16,27].

Hookworm infection was found to be the predominant helmenthic infection (2.1%). The finding was lower than the study conducted in Gonder (5.9%) [20], in Tanzania (9.0%) [12] and in China (4.6%) [6].

The second common helmenthic infection in study participants was *Ascaris lumbricoides* (1.2%) in PTB patients. The result is lower than the prevalence reported from meta-analysis in Iran (6%) [26], and higher than reported from China (0.5%) [6]. This difference may be due to socio-economic status and personal hygiene like fingernail status that statistically associated with intestinal parasite in our study.

We found that in our study, *Entamoeba histolytica/moshkovskii* was the major intestinal parasitic protozoan infection (8.2%). This result is concordant with study done by Tegegne et al in Gonder, Ethiopia (5.46%) but it is higher than a study carried out in China (1.4%)[6].

The present study suggests that age group of 20–59 years were most commonly affected by intestinal parasites. However, this finding is not in agreement with study finding reported by Panigrahi et al that intestinal parasitic infection (IPIs) was higher in age group 11–20 years in Odisha, India. The reason might be due to occupational related exposures of this age group [18].

In low and middle-income countries, TB and IPIs have been considered as public health issues. In present study, the overall magnitude of intestinal parasite and TB co-infection among smear positive was 25%. The finding is slightly lower than the study conducted in India (30.95%) [18] and another study conducted by Martha A. et al (33.3%) in Gondar University Hospital, Ethiopia [28].

Among the AFB positive patients Hookworm infection (5.6%) was predominantly detected. The result of this study is in agreement with result reported by Martha A.et al in Gondar University Hospital, Ethiopia [28]. Hookworm which is soil transmitted helminthic infection also reported from previously conducted studies on TB infected patients [1,11,19,20,26]. Evidences show that there is coincident in areas with high Hookworm endemicity and susceptibility to TB due to Hookworm has immunomodulation effect on M. *tuberculosis*. The co-infection inhibits protective effect of Th1 and Th17 cytokines and results in development of active TB [11,14,15].

We found that in current study the overall magnitude of AFB positive patients were 14.9%. The result of this finding was consistent with study done in Gonder, Ethiopia [20]. However, it was lower than similar studies carried out in Tanzania (81.2%) and South India (54%).This difference was also due to difference in diagnostic techniques. Both Purified Protein Derivative (PPD) and culture were performed in South India whereas ZN microscopy and culture was used in Tanzania [12,18].

In the present study, multivariate analysis showed that, both the residency area and fingernail status were factors significantly associated with intestinal parasites. Logistic regression analysis showed that the odds of contracting intestinal parasitosis among people who dwell in the rural area increases by a factor of 3.19 as compared to those who live in urban. This association implicated that residency area as a significant source of parasite infection in study area. Similar study done by Alemu *et al*. in Arba Minch, Ethiopia explained the same report [16]. Logistic regression analysis also indicated that compared to those who trimmed their fingernail, those who had untrimmed fingernail had higher odds of developing intestinal parasitosis.

Finding from this analysis showed that the odds of contracting PTB among participants who had contact history of TB patients increases by a factor of 2.88 as compared to those who did not. Similar report was obtained from the study conducted in Haramaya District, Eastern Ethiopia [7] and Malaysia in which contact of greater than 18hours with TB individual were statistically significantly associated with TB [9].

This study has some limitation like using of a single stool sample where the sensitivity could be lowered and modified Kato-Katz thick smears a semi-quantitative fecal examination technique for detection of helminthic ova was not used. Convenient sampling technique which not indicated the normal representativeness was used to select sample units.

## Conclusion

Although a single sample per study participant was performed for stool examination, the prevalence of intestinal parasites and tuberculosis among PTB suspected patients was high, indicating that they are still a serious public health problem. Hookworm infection was found to be the predominant helmenthic infection followed by *Ascaris lumbricoides*. The co-infections were observed between Protozoa and PTB as well as Helmenths and PTB that may exacerbate the severity of disease. People who dwell in rural area and untrimmed fingernail status were at significant risk in acquiring intestinal parasitic infections. Having contact history of TB patients was statistically significantly associated with PTB. It is important to consider screening TB patients for intestinal parasites and treat co-infection properly.
